# Galectin-3 Induces Clustering of CD147 and Integrin-β1 Transmembrane Glycoprotein Receptors on the RPE Cell Surface

**DOI:** 10.1371/journal.pone.0070011

**Published:** 2013-07-29

**Authors:** Claudia S. Priglinger, Christoph M. Szober, Siegfried G. Priglinger, Juliane Merl, Kerstin N. Euler, Marcus Kernt, Gabor Gondi, Jennifer Behler, Arie Geerlof, Anselm Kampik, Marius Ueffing, Stefanie M. Hauck

**Affiliations:** 1 Department of Ophthalmology, Ludwig-Maximilians-University, Munich, Germany; 2 Department of Ophthalmology, Allgemeines Krankenhaus der Stadt Linz, Linz, Austria; 3 Research Unit Protein Science, Helmholtz Center Munich, German Research Center for Environmental Health (GmbH), Neuherberg, Germany; 4 Research Unit Gene Vectors, Helmholtz Center Munich, German Research Center for Environmental Health (GmbH), Neuherberg, Germany; 5 Protein Expression and Purification Facility, Institute of Structural Biology, Helmholtz Center Munich, German Research Center for Environmental Health (GmbH), Neuherberg, Germany; 6 Centre of Ophthalmology, Institute for Ophthalmic Research, University of Tübingen, Tübingen, Germany; Eye Hospital, Charité, Germany

## Abstract

Proliferative vitreoretinopathy (PVR) is a blinding disease frequently occurring after retinal detachment surgery. Adhesion, migration and matrix remodeling of dedifferentiated retinal pigment epithelial (RPE) cells characterize the onset of the disease. Treatment options are still restrained and identification of factors responsible for the abnormal behavior of the RPE cells will facilitate the development of novel therapeutics. Galectin-3, a carbohydrate-binding protein, was previously found to inhibit attachment and spreading of retinal pigment epithelial cells, and thus bares the potential to counteract PVR-associated cellular events. However, the identities of the corresponding cell surface glycoprotein receptor proteins on RPE cells are not known. Here we characterize RPE-specific Gal-3 containing glycoprotein complexes using a proteomic approach. Integrin-β1, integrin-α3 and CD147/EMMPRIN, a transmembrane glycoprotein implicated in regulating matrix metalloproteinase induction, were identified as potential Gal-3 interactors on RPE cell surfaces. In reciprocal immunoprecipitation experiments we confirmed that Gal-3 associated with CD147 and integrin-β1, but not with integrin-α3. Additionally, association of Gal-3 with CD147 and integrin-β1 was observed in co-localization analyses, while integrin-α3 only partially co-localized with Gal-3. Blocking of CD147 and integrin-β1 on RPE cell surfaces inhibited binding of Gal-3, whereas blocking of integrin-α3 failed to do so, suggesting that integrin-α3 is rather an indirect interactor. Importantly, Gal-3 binding promoted pronounced clustering and co-localization of CD147 and integrin-β1, with only partial association of integrin-α3. Finally, we show that RPE derived CD147 and integrin-β1, but not integrin-α3, carry predominantly β-1,6-N-actyl-D-glucosamine-branched glycans, which are high-affinity ligands for Gal-3. We conclude from these data that extracellular Gal-3 triggers clustering of CD147 and integrin-β1 via interaction with β1,6-branched N-glycans on RPE cells and hypothesize that Gal-3 acts as a positive regulator for CD147/integrin-β1 clustering and therefore modifies RPE cell behavior contributing to the pathogenesis of PVR. Further investigations at this pathway may aid in the development of specific therapies for PVR.

## Introduction

It is well established that ligand binding and cell surface cross-linking of transmembrane proteins can lead to the assembly of large multicomponent protein complexes [1]–[3]. While in this respect protein-protein interactions have been well studied in the recent years, there is an increasing awareness that ligand binding to information stored in cell surface glycans can also lead to the assembly of large component protein complexes and modulate transmembrane signaling [4], [5]. Figuring prominently in deciphering the information stored in the glycan complexes is the protein family of galectins. Galectins belong to the large family of lectins which bind to oligosaccharide complexes specifically via beta (β)-galactoside moieties. Among these the 30 kDa member Galectin-3 (Gal-3) is unique in that it is composed of a C-terminal carbohydrate recognition domain and an N-terminal non-carbohydrate-binding domain that facilitates its multimerization [6]. Gal-3 has been shown to function through both intracellular and extracellular actions. Related to its intracellular functions, Gal-3 has been identified as a component of heterogeneous nuclear ribonuclear protein (hnRNP) [7], a factor in pre-mRNA splicing [8], and has been found to control cell cycle and prevent T cell apoptosis [9], whereas extracellular Gal-3 has been demonstrated to function in activating various types of inflammatory cells or mediating cell-cell and cell-extracellular matrix interactions [2], [10], [11].

Because of its ability to multimerize via its N-terminal domain and bind specific carbohydrate branches by the C-terminal domain, Gal-3 is thought to cross-link glycoproteins on the plasma membrane and form a cell surface molecular lattice [12]. In this respect there is an increasing awareness in the literature that Gal-3 has a fine specificity for β-1,6-N-actyl-D-glucosamine (GlcNAc) branched glycans [13]–[15] and that sufficient Gal-3 binding to glycoproteins is critically dependent on the presence of specific oligosaccharides and complex glycan constellations in the vicinity of β-galactose (reviewed by Brewer) [16].Gal-3 has been found to associate with many cell surface molecules and the number of ligands identified is still likely to grow: these include carcinoembryonic antigen (CEA), MUC1, lysosomal-membrane-associated glycoproteins (LAMPs)-1 and -2, Mac-1 and Mac-3, CD98, CD45, CD71 [2], [17], [18], and the glycosylated transmembrane receptors for epidermal growth factor (EGF), transforming growth factor beta (TGF-β), or vascular endothelial growth factor (VEGF) [12], [14], among others. Although the functional relevance of these interactions is not known in all cases, it has been found that association of the cell surface glycoproteins CD45 and CD71 with Gal-3 triggers T-cell apoptosis [2]. Binding of Gal-3 to VEGF-R2 retains the receptor on the plasma membrane of endothelial cells thereby promoting VEGF and basic fibroblast growth factor (bFGF)-driven angiogenesis and interaction of Gal-3 with proteins from the integrin family of transmembrane receptors has been found to regulate integrin-dependent cell adhesion, spreading, and migration: in endothelial cells association of Gal-3 with alpha(α)v beta(β)3 integrin induces clustering of the receptor and activates the signaling pathways that promote endothelial cell migration in the angiogenesis cascade [10], [12]. Binding of Gal-3 to α5β1 integrin promotes the α5β1 integrin dynamics associated with carcinoma cell motility [13], and Gal-3 induced cross-linking of α3β1 integrin induces lamellipodia formation in corneal epithelial cells [19].

Epithelial-to-mesenchymal transition, attachment, spreading and migration of retinal pigment epithelial cells with a myofibroblastic phenotype are believed to be the key events in the pathogenesis of proliferative vitreoretinopathy (PVR) [20], [21]. Despite of incessant advances in vitreoretinal surgery PVR is the leading cause for treatment failure in retinal detachment surgery. Clinically, PVR is characterized by formation of epi- and subretinal fibrocellular membranes, which contract and lead to repetitive retinal detachment and ultimately loss of vision. In the onset of the disease the break-down of the blood retinal barrier as a result of retinal injury leads to an inflammatory reaction with the release of inflammatory cytokines and growth factors, including tumor necrosis factor (TNF)-α, interleukin-6 (IL-6), interleukin-1β (IL-1β), TGF-β, hepatocyte growth factor, platelet derived growth factor or fibroblast growth factor (FGF), among others [22]–[24] into the vitreous, which contribute to the epithelial-to-mesenchymal transition of the retinal pigment epithelial cells (RPE) and drive the formation of PVR membranes. The establishment of PVR is critically dependent on the adhesive, migratory, and matrix remodelling behaviour of these cells. In the past integrins gained increasing attention in this respect and several studies have identified integrins αvβ3, αvβ5, integrin α subunits 2,3,4,5,6,v, and integrin β subunits 1,2,3 on RPE cells and cells within PVR membranes [25]–[27]. Traditionally, the activation of integrins in PVR has been investigated in the context of protein-protein interactions such as only recently the activation of α5 integrin in RPE cells by epidermal growth factor (EGF) [28]. However, the role of integrin glycans in modulating integrin clustering and the existence of modifying factors in the RPE have not been investigated so far. In a previous study we noted an upregulation of Gal-3 in dedifferentiated RPE cells and were able to substantially inhibit attachment and spreading of RPE cells by recombinant Gal-3. Exogenous Gal-3 bound to the RPE cell surface in a carbohydrate-dependent manner and exerted its effect, at least in part, by interfering with adhesion-related extracellular signal regulated kinase (ERK)-dependent signaling, suggesting the presence of a cell surface glycoprotein receptor [29]. The identity of Gal-3 ligands on the RPE cell surface however remains unknown and as galectin-mediated functions and ligand interactions can vary with the cell type under study, findings from one cell type cannot readily be extrapolated to another one [30]–[32]. Identification of a receptor for Gal-3 on RPE cells may therefore advance understanding of cellular processes associated with RPE epithelial-to-mesenchymal transition and the pathogenesis of proliferative vitreoretinal disorders, as well as aid in the development of specific therapies.

To identify and characterize RPE-specific cell-surface glycoproteins that might form complexes with Gal-3 we started off with co-immunoprecipitation followed by a proteomic approach to identify Gal-3 containing glycoprotein complexes. The findings were confirmed by reciprocal immunoprecipitation, fluorescence activated cell sorting (FACS) analysis, and immunohistochemical co-localization. Furthermore, the status of β1,6 N-glycosylation of dedifferentiated RPE cells and the candidate glycoprotein ligands were determined. By applying this approach we have discovered a novel interaction between Gal-3, the integrin-β1 subunit and the CD147 protein. The latter molecule is a widely distributed cell-surface glycoprotein with abundant expression at the basolateral and apical surfaces of differentiated RPE that in healthy RPE may play a role in targeting of monocarboxylate transporters to the plasma membrane [33], [34].

## Materials and Methods

### Isolation of Human RPE Cells and Human RPE Cell Culture

RPE cells were isolated from human cadaver eyes obtained from the Eye Bank of the Department of Ophthalmology at the Linz General Hospital (Linz, Austria) and processed within four to 24 h after death. Donor age ranged from 27 to 79 years and none of the donors had a known history of eye disease. Methods for securing human tissue were humane, included proper consent and approval of the relatives, and complied with the Declaration of Helsinki. Human postmortem donor eyes were enucleated by an ophthalmologist according to the institutiońs standard operating procedures. The isolation of RPE cells from human cadaver eyes for scientific purposes was approved by the ethical committee of the Land Oberoesterreich. Human retinal pigment epithelium (RPE) cells were harvested from twenty post-mortem eyes following the procedure as described previously [35], [36]. Primary RPE cells were subcultured and maintained in Dulbeccós modified Eagles medium (DMEM; Biochrom, Berlin, Germany) supplemented with 10% fetal calf serum (FCS; Biochrom) at 37°C and 5% CO_2._ Primary human RPE cells of passage 3 to 7 were used for experiments. When indicated, different concentrations of galectin-3 (Gal-3) were included in the culture medium. For preparation of protein lysates the posterior poles from eyes of seven different donors were prepared as described above. RPE cells were released from Bruch’s membrane by gently pipetting ice cold phosphate buffered saline (PBS, pH 7.4) solution into the eye cup. The suspended RPE cells were transferred to a 35 mm^2^ petri dish and checked for cross contamination using a microscope. Cell suspensions were then transferred to a 2.0 ml microcentrifuge tube and centrifuged for 5 min at 800 rpm. After centrifugation the supernatant was removed and replaced by RIPA cell lysis buffer (50 mM Tris, pH 8.0; 150 mM NaCl; 1% NP40; 0.5% deoxycholate; 0.1% SDS) containing an appropriate amount of protease inhibitors (Complete Mini, Roche, Mannheim, Germany). Further protein extraction was carried out as described above. Cell preparations were snap frozen in liquid nitrogen and stored at −70°C for future use.

### Tissue Samples

Samples of PVR membranes were obtained from patients who were undergoing vitreoretinal surgery for PVR following rhegmatogenous retinal detachment. Before surgery, each participant gave its informed written consent. The procedure was documented in accordance with the guidelines of the institutional review board of the Department of Ophthalmology at the Ludwig-Maximilians-University Munich (Reference Number: IRB-CP-00142009) and was conducted according to the declaration of Helsinki. The collection of PVR membranes for scientific purposes was approved by the ethical committee of the Klinikum of the Ludwig-Maximilians-University Munich (Permit Number: 331-09) and the ethical committee of the Land Oberoesterreich. Operations (n = 7) were carried out at the Department of Ophthalmology of the Ludwig-Maximilians-University (Munich, Germany) and at the Department of Ophthalmology, AKH Linz (Linz, Austria) by different surgeons, who used conventional three-port vitrectomy. Epi- and subretinal membranes were separated from the retina by peeling whole tissues. During the operation membranes were put into phosphate buffered saline (PBS, pH 7.4), then transferred to 4% formaldehyde and processed for embedding in paraffin.

### Human Galectin-3: Expression, Purification, Labeling and Quality Controls

Human Gal-3 was cloned in the bacterial pETM-11 expression vector using restriction enzymes NcoI/HindIII, resulting in the fusion of the gene to an N-terminal His_6_-tag. pETM-11/hgalectin3 was transformed into the *E. coli* strain BL21 (DE3) and cultured at 20°C in 2-L flasks containing 500 ml ZYM 5052 auto-induction medium [37] and 30 µg/ml kanamycin. Cells were harvested by centrifugation after reaching saturation, resuspended in 30 ml lysis buffer (50mM Tris-HCl, 300mM NaCl, 20mM imidazole, 10mM MgSO_4_, 10 mg/ml DNaseI, 1mM AEBSF.HCl (serine protease inhibitor), 0.2% (v/v) NP-40, 1 mg/ml lysosyme, 0.02% (v/v) 1-thioglycerol, pH 8.0), and lysed by sonication. The lysate was clarified by centrifugation (40,000×g) and filtration (0.2 µM). The supernatant was applied to a 5-ml HiTrap Chelating HP column (GE Healthcare, Munich, Germany), equilibrated in buffer A (50mM Tris-HCl, 300mM NaCl, 20mM imidazole, 0.01% (v/v) 1-thioglycerol, pH 8.0) using an Äkta Purifier (GE Healthcare). The column was successively washed with buffer A, buffer B (similar to buffer A with additional 1M NaCl), and buffer C (similar to buffer A containing additional 50mM imidazole) until a stable baseline was reached (monitored at 280nm). Bound proteins were eluted with buffer D (50mM Tris-HCl, 300mM NaCl, 300mM imidazole, 0.01% (v/v) 1-thioglycerol, pH 8.0) and fractions containing protein pooled and dialyzed overnight at 4°C against 1 L buffer A in the presence of His_6_-tagged tobacco etch virus (TEV) protease in a 1∶50 molar ratio (TEV:protein). TEV-cleaved protein was further purified by affinity chromatography as described above, and the flow-through and protein containing wash fractions were pooled and concentrated to less than 5 ml. This was subsequently subjected to size exclusion chromatography using a HiLoad 16/60 Superdex 75 column (GE Healthcare), equilibrated in buffer E (50mM Tris-HCl, 300mM NaCl, and 0.01% (v/v) 1-thioglycerol, pH 8.0). The main elution peak containing galectin-3 was collected and stored at 4°C.

Purity of Gal-3 was confirmed by mass spectrometry. For biotinylation, 2 mg of purified Gal-3 were dialyzed overnight at 4°C against 0.1M sodiumhydrogencarbonate; pH 8.0, followed by a 1 hour dialysis against the same buffer, but with pH 9.2. Gal-3 was then biotinylated for 1 hour at RT with 200 µg biotinamidohexanoic acid N-hydroxysuccinimide ester according to the manufactureŕs instructions (Sigma, Taufkirchen, Germany) followed by dialysis against PBS overnight. Biotinylation was confirmed by western blot analysis by detection with HRP-coupled streptavidin (Roche, Mannheim, Germany).

### Isolation of Galectin-3 Binding Proteins in Human RPE Cells by Co-immunoprecipitation

Human RPE cells grown in 75 cm^2^ tissue culture flasks were washed twice with ice-cold PBS, collected, and lysed at low stringency conditions in 1% Triton X-100 buffer to avoid dissociation of the protein complexes. For immunoprecipitation experiments used for protein complex identification by mass spectrometry, RPE cells were after washing in PBS reacted with 2 mM DTSSP (3,3′-dithiobis[sulfosuccinimidylpropionate]; Sigma) followed by addition of 20mM Tris pH 7.5 for a 15 minute quenching reaction and one wash in PBS before cell lysis. For complete lysis the cellular extracts were allowed to incubate in the presence of an appropriate amount of proteinase inhibitor cocktail (Complete Mini™; Roche) on a rotational shaker for 1 hour at 4°C. After centrifugation for 30 minutes at 16,000 g in the cold the protein content in the supernatant was determined using the Bradford protein assay reagent (Biorad, Munich, Germany). The solubilized protein fraction was then incubated with ProteinG Sepharose beads (ProteinG Sepharose4 Fast Flow beads™, GE Healthcare) in equilibration buffer (50 mM Tris, pH 7.2; 150 mM NaCl) for 1.5 hours at 4°C. The supernatant was then collected and 375 µg of precleared RPE cell lysate was incubated with ProteinG sepharose beads in a total volume of 750 µl in the presence of 2 µg monoclonal anti-human Gal-3 (Novocastra, Newcastle, UK), or monoclonal anti-human CD147 (Novus Biochemicals, Cambridge, UK), or monoclonal anti-human integrin-α3 (MCA5694T; AbD Serotec, Oxford, UK) for 2 hours on a rotational shaker in the cold. As appropriate negative control in parallel experiments cellular lysates were incubated with ProteinG™ beads in the presence of isotype-matched, pre-immune mouse IgG. The suspension was then transferred to microspin columns (GE-Healthcare), washed several times with 1% Triton X-100 buffer, and bound protein complexes were eluted with 2x Laemmli buffer. The immunoprecipitated complexes were then gently denatured at 70°C for 5 minutes, centrifuged at 1,000×g for 2 minutes and the supernatant was further processed for protein identification using mass spectrometry or subjected to SDS PAGE. Each co-immunoprecipitation was repeated at least eight times using RPE cell lines derived from different human donors.

### Mass Spectrometric Protein Identification and Quantification

For mass spectrometry, eluates were precipitated with chloroform/methanol, dissolved in ammonium bicarbonate buffer supplemented with Rapigest (Waters, Milford, MA, USA), followed by reduction using dithiothreitol (Merck, Darmstadt, Germany), and alkylation in iodoacetamide (Merck). Proteins were then subjected to tryptic digest as described before [38] and peptide samples were acidified with TFA to a final concentration of 5% to precipitate the Rapigest surfactant. For LC MS/MS analysis peptides were loaded automatically on a trap column and separated by HPLC on an UltiMate nano-LC-system (LC Packings, Bensheim, Germany) on an analytical column with a 120 min gradient at 300 nL/min directly into the mass spectrometer (LTQ Orbitrap XL, Thermo Fisher Scientific, Schwerte, Germany). From the MS prescans, ten most abundant peptide ions were selected for fragmentation and during fragment analyses, high resolution MS spectra were acquired with a mass range from 200 to 1500 m/z.

For relative quantification, the acquired spectra were loaded into the Progenesis LC-MS software (version 2.5, Nonlinear Dynamics, Newcastle Upon Tyne, UK) for label free quantification and analyzed as described previously [38], [39]. Briefly, after feature detection and exclusion of features with only one charge or more than eight charges, all raw abundances were normalized across samples to minimize variation introduced by experimental variations and then MS/MS spectra were exported as Mascot generic file (mgf). Peptide identification was performed with Mascot search engine (version 2.2, Matrix Science Inc., Boston, MA, USA) in the Ensembl Human database (Release 69, October 2012; 100607 sequences) with 10 ppm mass tolerance for peptides and 0.6 Da mass tolerance for fragments ions, carbamidomethylation set as fixed modification and oxidation of methionines and deamidations of asparagines and glutamines allowed as variable modifications. A Mascot-integrated decoy database search calculated a false discovery rate of <1.5%, using an ion score cut-off of 30 and a significance threshold of p<0.01 for all searches. Peptide assignments were re-imported into the Progenesis software. After summing up the abundances of all peptides allocated to each protein, the ratios of each protein as compared to the non-immune mouse IgG negative controls were calculated based on these abundances. All identified proteins with their respective raw and normalized abundances are listed in table S1.

### Western Blot Analysis

Immunoprecipitated proteins were separated on SDS-PAGE gels. After wet blotting onto a polyvinyl difluoride membrane (GE Healthcare), membranes were blocked in 5% nonfat dry milk and then incubated with mouse monoclonal anti-human Gal-3 (Novocastra, 1∶250), or mouse monoclonal anti-human CD147 (Novus Biochemicals, 1∶1000), or a goat polyclonal anti-human integrin-α3 (SantaCruz, 1∶500), or a mouse anti-human integrin-β1 (BD Biosciences, 1∶1000) in 5% skim milk in TBS-T (0,1% Tween 20, 50mM Tris pH 7.4, 5mM NaCl) and allowed to react overnight at 4°C. Appropriate secondary horseradish peroxidase-coupled antibodies (JacksonImmunoResearch, Newmarket, UK) were used in a dilution of 1∶15000. Protein signals were then visualized using ECL Plus enhanced chemiluminescence kit (GE Healthcare) and signals were captured on Hyperfilm ECL (GE Healthcare). All experiments have been repeated at least six times using RPE cells derived from different donors.

### Immunocytochemistry and Immunohistochemistry of RPE Cell Cultures

RPE cells were grown on glass coverslips and maintained in DMEM supplemented with 10% FCS until they reached subconfluence. When indicated, cells were then treated with galectin-3 (40 µg/mL) in DMEM for 30 minutes at 37°C. After three washes in PBS, cells were fixed in 4% paraformaldehyde for 5 minutes on ice. After another three washes in PBS, specimen were blocked with 1% BSA in PBS for 1 hour at room temperature, and incubated with polyclonal rabbit anti-human Gal-3 (dilution 1∶100; Santa Cruz, Heidelberg, Germany), and monoclonal mouse anti-human CD147 (dilution 1∶100; Novus Biochemicals), and either monoclonal rat anti-human integrin-α3 (dilution 1∶100; dilution 1∶100; clone 8G7, see below), or a monoclonal rat anti-human integrin-β1 (dilution 1∶50; clone AIIB2; developed by C.H. Damsky and obtained from the Developmental Studies Hybridoma Bank developed under the auspices of the NICHD and maintained by The University of Iowa, Department of Biology, Iowa City, USA) for 2 hours at RT. After three washes in PBS nuclei were counterstained with 1 µg/mL Hoechst 33342 (Sigma) and cells were incubated with Alexa-Fluor 488™-conjugated anti-mouse IgG, or Alexa-Fluor 568™-conjugated anti-rabbit IgG, or Alexa-Fluor 647™-labelled anti-rat IgG, or the respective combinations as secondary antibodies for 1 hour at RT in the dark (diluted 1∶2,000; -all obtained from Invitrogen, Karlsruhe, Germany). All antibodies were diluted in PBS containing 1% BSA. Cells incubated with non-related isotype IgG or secondary antibodies alone served as negative controls. Images were generated on a fluorescence microscope using the apotome mode (Axio Imager Z1Zeiss, Göttingen, Germany) and the Axio Vision 4.6 software (Zeiss). Monoclonal antibody 8G7 (rat IgG2a) against integrin-α3 (CD49c) was generated by injecting cell membrane particles (exosomes) of the human lung cancer cell line A549 into Lou/c rat. The fusion was performed using standard procedures. Hybridoma supernatants were tested by flow cytometry on A549 cells. The specificity of the mAb was determined by immunoprecipitation followed by mass spectrometry.

### Flow Cytometry

Measurement of RPE cell surface expression of candidates was performed on FACS Canto II with FACS Diva 6.1.3 software (both BD Biosciences, Heidelberg, Germany). Cell staining was performed in 96 well round-bottom plates with 2×10^5^ cells per well. Cells were either incubated with biotinylated recombinant Gal-3 (50 µg/mL) or a monoclonal anti-human CD147 antibody (dilution 1∶10; Novus Biochemicals) or a monoclonal anti-human integrin-α3 antibody (clone 8G7, dilution 1∶5) or a monoclonal anti-human integrin-β1 (clone JB1A, Chemicon, dilution 1∶50) for 1 hour at 4°C. Biotinylated Gal-3 was then stained with avidin-FITC (dilution 1∶200; Southern Biotech, Birmingham, Alabama, USA), CD147 and integrin- β1 with secondary goat anti-mouse IgG1 PE antibody (dilution 1∶300; Southern Biotech) and integrin-α3 with secondary mouse anti-rat IgG2a FITC-antibody (dilution 1∶200; TIB173; ATCC, Wesel, Germany) in staining buffer for 30 min at 4°C. Cells were kept at 4°C in staining buffer with 1% PFA until processing. 10000 cells were measured per staining. For blocking experiments, cells were first incubated with anti-human CD147, anti-human integrin-β1 or anti-human integrin-α3 antibody as described above to block CD147, integrin-β1 and integrin-α3 sites on the cell surface, followed by incubation with 50 µg/mL biotinylatedGal-3 for 1 hour at 4°C and avidin-FITC as described. Isotype controls were performed for Gal-3 and all antibodies used, respectively. In order to evaluate the carbohydrate dependence of Gal-3, binding cells were pre-incubated with 100 mM β-lactose (Sigma), the haptenic sugar to block carbohydrate-dependent galectin-binding, or 100 mM of the non-specific sugar sucrose for 20 minutes before Gal-3 was added.

### Lectin Affinity Analyses

Protein (600 µg) from RPE cell lysates prepared as described above in RIPA buffer (50 mM Tris-HCl pH 7,4; 150 mM NaCl; 0.1%(w/v) SDS; 0.5% (w/v) sodium deoxycholate; 1% (v/v) NP-40) was precleared for 1 hour with 15 µl of ProteinG agarose beads (SantaCruz Biotechnology) to reduce non-specific binding, and then incubated with 15 µl agarose-conjugated PHA-L, or agarose-conjugated ConA, or beads alone (all obtained from Vector Laboratories, Burlingame, CA, USA) for 1 hour at 4°C on a rotational shaker. The beads were washed 3 times in RIPA cell lysis buffer and once in PBS. Bound proteins were eluted by boiling in SDS-PAGE sample buffer without β-mercaptoethanol, separated by 12% SDS-PAGE, transferred to PVDF membranes, and analyzed by western blot analysis using the same antibodies as described above.

### Immunohistochemical Staining of Tissue Sections

For immunohistochemistry PVR membranes were fixed in 4% formaldehyde, embedded in paraffin and cut into sections of 8 µm each. Sections were then set onto glass slides and incubated at 42°C for 48h to prevent washing off the sections during the process of immunohistochemical staining. In order to remove the paraffin and to rehydrate the sections, they were first rinsed with xylene (once for 5 min) and isopropanol (twice for 5 min), followed by ethanol (96% and 70% - each for 5 min) and finally millipore water (5 min). Heat antigen retrieval of Gal-3 was performed by boiling the sections in citrate buffer pH 6.1 (Dako, Hamburg, Germany) for 15 min. Prior to incubation with the primary antibody, sections were blocked for 40 min in a humid environment with 1% BSA in phosphate buffered saline–tween (PBS +0.1% Tween20), in order to prevent non-specific staining. Sections were then incubated with biotinylated Gal-3 (5 µg/ml diluted in PBS-T +1% BSA) or anti-human CD147 (1∶100, Novus Biochemicals), or anti-human integrin-β1 (dilution 1∶50; Developmental Studies Hybridoma Bank, clone AIIB2) overnight at 4°C followed by incubation with Avidin-FITC diluted in PBS-T +1% BSA (dilution 1∶200, Invitrogen, Darmstadt, Germany) or the respective secondary antibodies as described above for one hour at RT in the dark. Cell nuclei were counter-stained with 4′,6-diamidino-2-phenylindole (DAPI; dilution 1∶1000, Invitrogen). Finally, the sections were mounted with glass coverslips using fluorescence mounting medium (Dako). Fluorescent images were recorded with an Axio Imager M1 or Z1 and the Axio Vision 4.6 software (both from Zeiss, Göttingen, Germany) at a total magnification of 40fold.

## Results

### CD147 and Integrin-β1 are Major Gal-3 Binding Proteins in Cultured RPE Cells

In order to identify cell surface ligands specific for RPE cells we used cultured human RPE cells as a model system for early PVR. When human RPE cells are cultured on plastic, they escape growth arrest and fail to maintain a differentiated morphology. For this reason this provides a well-accepted *in vitro* model for the fibroblast-like phenotype of RPE cells as found in PVR [40]. Potential Gal-3 interacting proteins were isolated by co-immunoprecipitation experiments followed by identification with liquid chromatography coupled to mass spectrometry (LC-MS/MS).

In an attempt to find a receptor for exogenous Gal-3 on the cell surface we in a first step lysed RPE cells with prior addition of 12.5–25.0 µg/mL Gal-3. These concentrations are just above the bottom level of the dose response curve for inhibition of RPE attachment as determined in previous experiments [29] and were chosen in order to avoid oversaturation of ligands and non-specific binding. Despite of thorough washing before incubation with anti-Gal-3 coupled beads and a series of low stringency washes to avoid dissociation of the Gal-3 containing protein complexes subsequent mass-spectrometry detected an excess of Gal-3 in the eluate with qualitatively unreliable peptide identifications for candidate protein interactors (data not shown). For this reason we next co-immunoprecipitated endogenous Gal-3 from whole RPE cell lysates without exogenously added Gal-3. Mass spectrometric analysis of solubilized proteins that bound with high affinity to the sepharose-bound monoclonal anti-Gal-3 antibody resulted in the unequivocal identification of twenty five proteins (Table 1). Gene ontology (GO) annotations assigned thirteen (52%) to a nuclear or cytoplasmic location (hnRNP A3, 40s ribosomal protein S27-like protein, 60S ribosomal protein L8, UPF0027 protein C22orf28, nucleosome assembly protein 1-like 4, protein LSM12 homolog, nuclear fragile X mental retardation-interacting protein 2, interleukin enhancer-binding factor 3, nucleophosmin, POTE ankyrin domain family member E, RNA binding motif protein X-linked-like 1, histone H1.3, RNA-binding protein FUS), five (20%) to the cytoskeleton or structural components (septin-11, myosin-11, keratin 5, tropomyosin β-chain, gelsolin), two (8%) proteins were predominantly secreted (α2 macroglobulin, galectin-3 binding protein), and five (20%) proteins were attributed to the cell membrane (CD147, integrin α3, integrin β1, monocarboxylate transporter-4, galectin-3). The data are compiled in Table 1 (subcellular localization); biological functions of the candidate ligands are also included.

**Table 1:** Isolation of RPE cell proteins with high affinity binding to Gal-3.

Accession number	ratio Gal-3/neg control	Gene name	GO term “Plasma membrane or extracellular region”	Glycosylation	Protein name	Subcellular localization	Biological process
ENSP00000376309	Infinity	HNRNPA3			Heterogeneous nuclear ribonucleoprotein A3 (hnRNP A3)	nucleus	RNA trafficking and splicing
ENSP00000331019	Infinity	RPS27L			40S ribosomal protein S27-like protein	nucleus	DNA repair, apoptosis
ENSP00000333769	Infinity	BSG	X	X	CD147 (Basigin)	Golgi membrane, cell membrane, endoplasmatic reticulum	Tissue invasion, MMP-production, angiogenesis, targets MCTs to plasma membrane
ENSP00000264893	Infinity	SEPT11			Septin-11	cytoskeleton	Cytokinesis
ENSP00000262584	363.2	RPL8			60S ribosomal protein L8	cytoplasm	RNA binding, translation
ENSP00000216038	279.6	C22orf28			UPF0027 protein C22orf28	cytoplasm	RNA-binding
ENSP00000007722	124.3	ITGA3	X	X	Integrin alpha-3	cell membrane	Adhesion, migration, signalling
ENSP00000300036	54.2	MYH11			Myosin-11	cytosol, myosin filament	Stress fiber component, smooth muscle contraction
ENSP00000254301	48.5	LGALS3	X		Galectin-3	nucleus, cytoplasm, plasma membrane, secreted	Adhesion, migration, chemotaxis, differentiation
ENSP00000303351	39.2	ITGB1	X	X	Integrin beta-1	cell membrane	Adhesion, migration, signalling
ENSP00000436488	26.9	NAP1L4			Nucleosome assembly protein 1-like 4	cytoplasm	Nucleosome assembly
ENSP00000252242	26.0	KRT5			Keratin, type II cytoskeletal 5	intermediate filament	Structural component
ENSP00000354219	25.9	TPM2			Tropomyosin beta chain	cytoskeleton	Actin-filament binding, motility
ENSP00000293406	15.9	LSM12			Protein LSM12 homolog	nucleus, cytoplasm	RNA metabolism
ENSP00000340888	12.5	GSB			Gelsolin	cytoplasm, cytoskeleton	Actin-modulating protein
ENSP00000376150	11.0	SLC16A3	X		Monocarboxylate transporter 4	cell membrane	Energy metabolism
ENSP00000225388	10.9	NUFIP2			Nuclear fragile X mental retardation-interacting protein 2	cytoplasm, nucleus	RNA-binding
ENSP00000250241	10.7	ILF3			Interleukin enhancer-binding factor 3	cytoplasm, nucleus	Transcription
ENSP00000323929	8.9	A2M	X	X	Alpha-2-macroglobulin	cytosol, secreted	Protease inhibitor
ENSP00000296930	7.5	NPM1			Nucleophosmin	nucleus, nucleolus	RNA-binding, centrosome cycle
ENSP00000439189	7.2	POTEE			POTE ankyrin domain family member E	cytoplasm	ATP-binding
ENSP00000318415	5.2	RBMXL1			RNA binding motif protein, X-linked-like 1	cytoplasm	RNA-binding
ENSP00000244534	5.0	HIST1H1D			Histone H1.3	nucleus	Transcription, nucleosome condensation
ENSP00000254108	4.7	FUS			RNA-binding protein FUS	nucleus	DNA synthesis
ENSP00000262776	4.1	LGALS3BP	X	X	Galectin-3-binding protein	secreted	Cell adhesion, ECM component

As expected we in this approach predominantly found ligands for endogenously expressed Gal-3, consistent with the majority of the receptor proteins identified being assigned to the nucleus or cytoplasm. Based on our previous observation that exogenous Gal-3 binds to the RPE cell surface in a carbohydrate-dependent manner and thereby appears to influence RPE-cell adhesion our interest was focused on RPE-specific Gal-3 glycoprotein receptors on the RPE cell surface. Therefore proteins attributed to cytoskeleton, nucleus, or cytoplasm were excluded from further studies. Of the remainder all except for MCT-4 were described glycoproteins (Table 1) and therefore potential galectin-3 interacting proteins. Out of these all except for α2-macroglobulin, which is essentially located in the extracellular matrix (ECM), have been reported to play a role in cellular adhesion. Integrin-α3 and integrin-β1 have previously been shown to be binding partners of Gal-3 [19], validating the suitability of our approach.

The highest enrichment factor in the Gal-3 immunoprecipitation, however, together with a high Mascot confidence score (104) was observed for CD147, a transmembrane glycoprotein which has not previously been described in context with Gal-3. CD147 plays a role in several biological processes, including cell adhesion, plasma membrane integration of monocarboxylate transporters, inflammation, or angiogenesis [41], [42], among others. It is heavily glycosylated and may therefore be a reasonable ligand for Gal-3.

**Figure 1:**
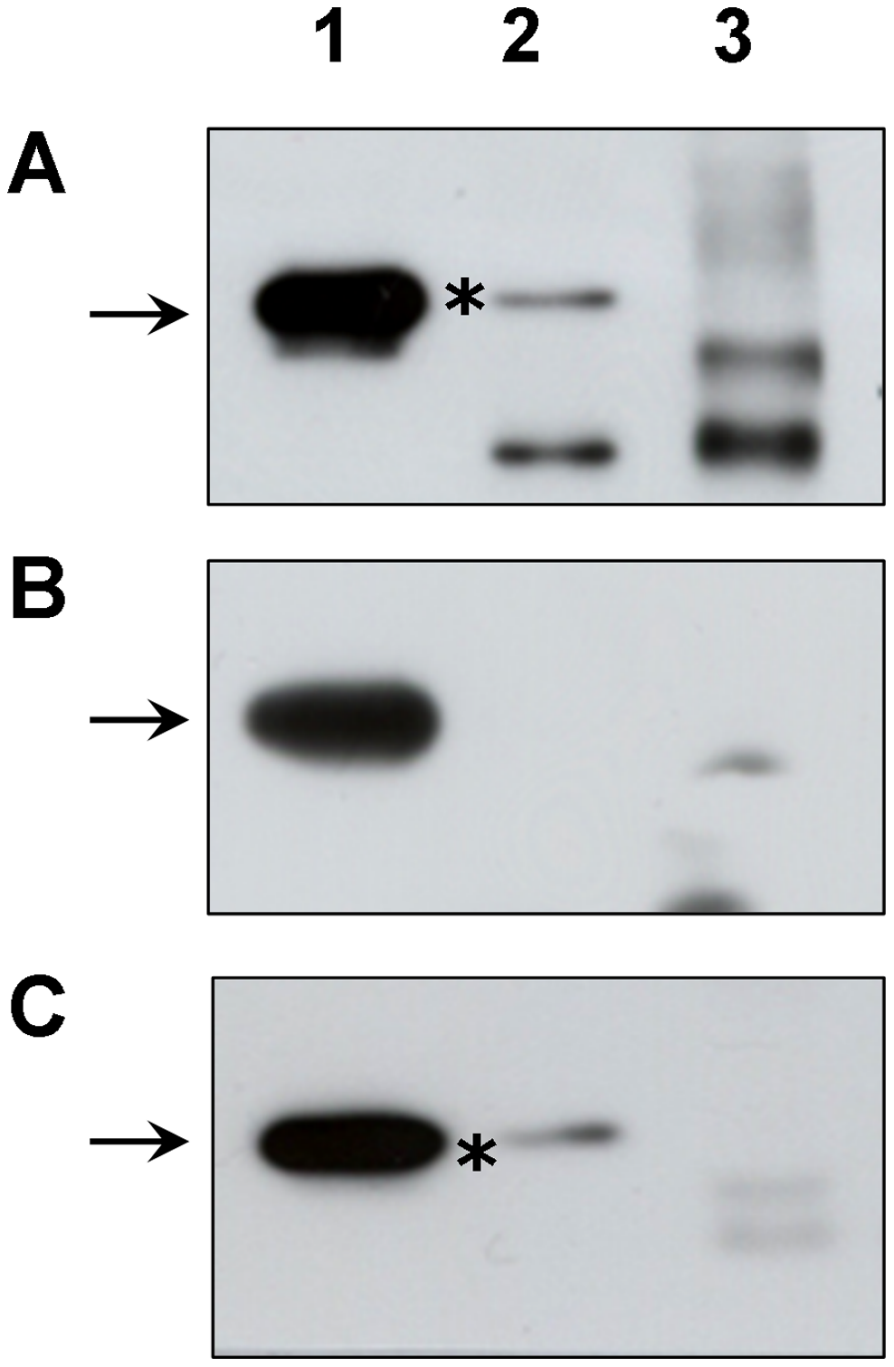
Gal-3 associates specifically with CD147 and integrin-β1 in cultured RPE cells. Whole cellular protein lysates were prepared by treatment of RPE cells with 1% Triton X-100 in PBS. (**A**) CD147 containing immunocomplexes were precipitated from 375 µg of proteins using 2 µg of a monoclonal anti-human CD147 antibody (*lane 2*), or a non-related isotype control (*lane 3*). Equal amounts of immunoprecipitation eluates, or whole cellular lysate (*lane 1*) were separated by SDS-PAGE and analyzed by immunoblotting using a monoclonal anti-Gal-3 antibody. (**B**, **C**) Cell lysates were processed as described for CD147 and precipitated using 2 µg of a monoclonal antibody recognizing specifically the integrin-α3 (B, *lane 2*) and integrin-β1 (C, *lane 2*) subunit, respectively. Note that the eluates precipitated with anti-CD147 (A; *lane 2*) and anti-integrin-β1 (C; *lane 2*) as well as the total cell extract (A,B; *lane 1*) contain the anti-Gal-3 reactive band at 30 kDa. In contrast, no anti-Gal-3 reactive component was recovered from cell extracts precipitated with anti-integrin-α3 (B; *lane 2*) and with the isotype control (A,B,C; *lane 3*). Experiments have been repeated at least eight times using cultured human RPE cells from different donors. Representative blots are shown.

In order to confirm the presence of Gal-3 in CD147 containing protein complexes reciprocal experiments were performed. Gal-3 immunoblotting confirmed the presence of the 30 kDa band representing Gal-3 (Fig. 1A, *lane 2*) in anti-CD147 immunoprecipitates, but not in the control immunoglobulin immunoprecipitates (Fig. 1A, *lane 3*). In parallel experiments integrin-α3 protein complexes were precipitated. To our surprise, western blot analysis failed to detect anti-Gal-3 reactive components in the eluted fraction from the integrin-α3 immunoprecipitation (Fig. 1B, *lane 2*), but Gal-3 could be recovered from re-immunoprecipitation experiments with an antibody specifically recognizing the integrin-β1 subunit (Fig. 1C, *lane 2*). These data suggest a probable interaction of Gal-3 with CD147 and integrin-β1 in cultured RPE cells, whereas the interaction with integrin-α3 may be rather indirect or less stable. This is also supported by the use of mild crosslinking before cell lysis in the mass spectrometry approach while the reverse immunoprecipitation experiments were performed without crosslinking.

### CD147, but not Integrin-α3, Serves as Major Galectin-3 Counter-receptor in Dedifferentiated RPE

**Figure 2:**
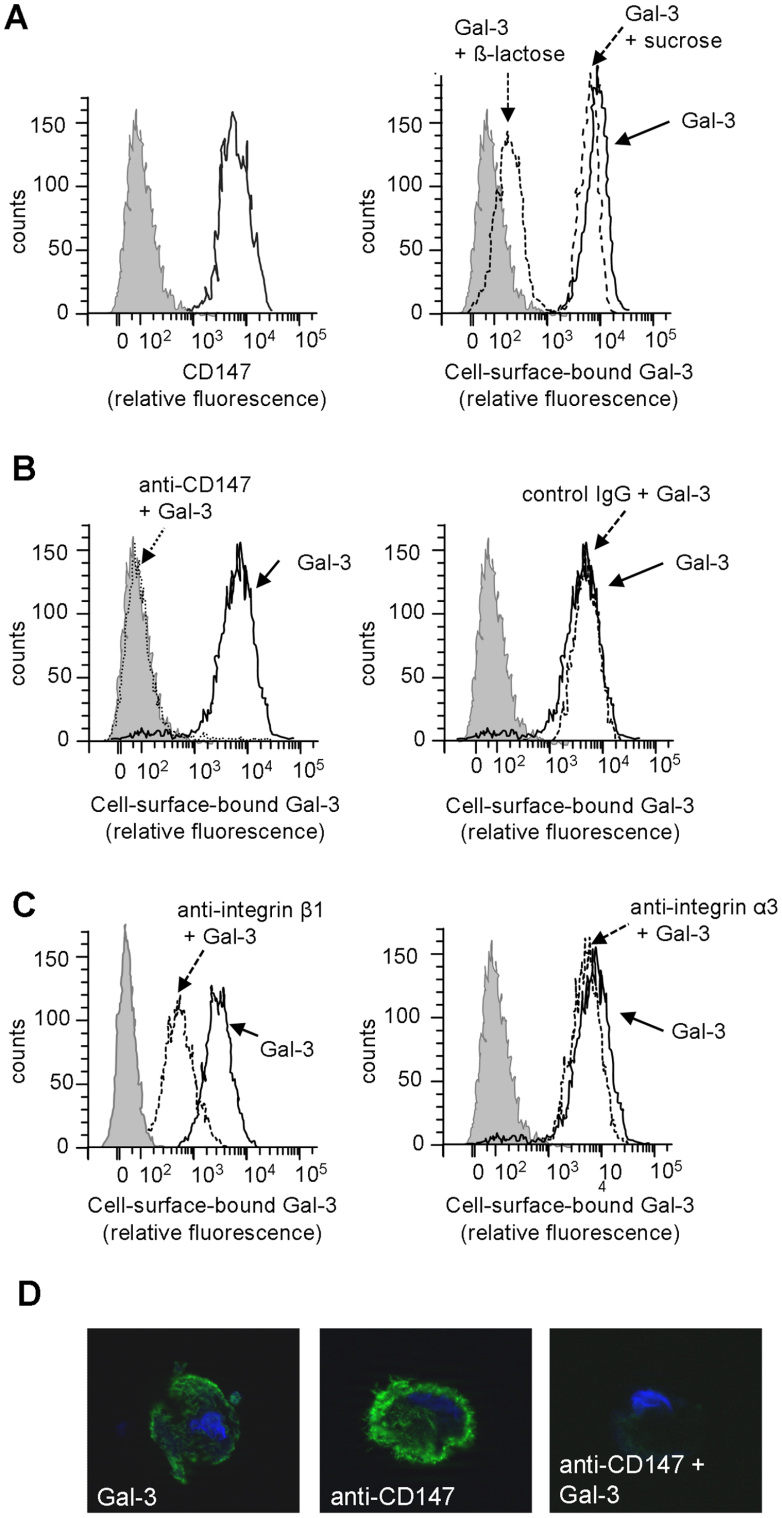
Interaction of Gal-3 with the RPE cell surface is mediated by carbohydrate-dependent binding to CD147 and integrin-β1, but not to integrin-α3. For monitoring the binding of recombinant Gal-3 to the RPE cell surface RPE cells were incubated with 40 ug/mL biotinylated Gal-3 and the cell surface binding was evaluated in a flow cytometer. The expression of CD147, integrin-β1 and integrin-α3 on the RPE cell surfaces was evaluated by staining with the respective antibodies, followed by measuring the relative fluorescence as described in the materials and methods section. (**A**) Staining with biotinylated recombinant Gal-3 and anti-CD147 confirms the presence of both proteins on the RPE cell surface. The interaction of Gal-3 with the RPE cell surface is mediated via its carbohydrate-binding domain, as it could be inhibited by adding 100 mM β-lactose but not by sucrose (*right panel*). (**B**) Pre-treatment of cells with a neutralizing anti-CD147 antibody reduces cell surface binding of Gal-3 to background levels (*left panel*). Incubation of cells with an isotype control IgG has no influence on Gal-3 cell surface binding (*right panel*). (**C**) Blocking of integrin-β1 partially reduces Gal-3 binding (*left panel*) while blocking of integrin-α3 binding sites fails to reduce Gal-3 binding to RPE cells (*right panel*). Relative fluorescence level of unstained cells incubated with streptavidin-FITC alone is also shown and represents the level of background fluorescence (shaded). (**D**) Fluorescence microscopy illustrates the absence of Gal-3 binding to the RPE cell surface when CD147 is blocked by treatment with a neutralizing antibody prior to the addition of Gal-3. The data are representative of three independent experiments with different RPE cell lines.

In order to further verify our findings from the co-immunoprecipitation experiment and to validate our novel Gal-3 binding protein CD147, flowcytometry as a measure for the nature of the interaction of exogenous Gal-3 and CD147 on the RPE cell surface was performed. This experiment provided us with five important results: first, we confirmed that both CD147 and exogenous Gal-3 are detected on the surface of dedifferentiated RPE cells (Fig. 2A). Second, co-incubation of cells with β-lactose, an inhibitor of carbohydrate-dependent Gal-3 binding but not with sucrose (used as osmolarity control), reduced Gal-3 binding to the RPE cell surface, confirming a carbohydrate-dependent Gal-3 cell surface interaction (Fig. 2A, *right panel*). Third, in support of CD147 being a direct Gal-3 interaction partner, binding of Gal-3 to RPE cells was substantially reduced when cells were pre-incubated with a neutralizing antibody to CD147 (Fig. 2B, *left panel*), but not by a non-relevant control IgG (Fig. 2B, *right panel*). Fourth, pre-incubation with a neutralizing antibody to integrin-β1 partially blocked binding of Gal-3 to the RPE cells (Fig. 2C, *left panel*) while blocking of integrin-α3 binding sites with neutralizing anti-integrin-α3 failed to reduce binding of Gal-3 (Fig. 2C, *right panel*). These findings further support the notion that CD147 is a major counter-receptor for Gal-3 on RPE cells, and that binding is also promoted by integrin-β1, while integrin-α3 may be rather an indirect receptor for Gal-3. Fluorescence micrographs illustrating the blocking of the Gal-3 binding sites by anti-CD147 are also presented (Fig. 2D).

### CD147 Co-localizes with Gal-3 and Integrin-β1 on RPE Cells in Culture

**Figure 3:**
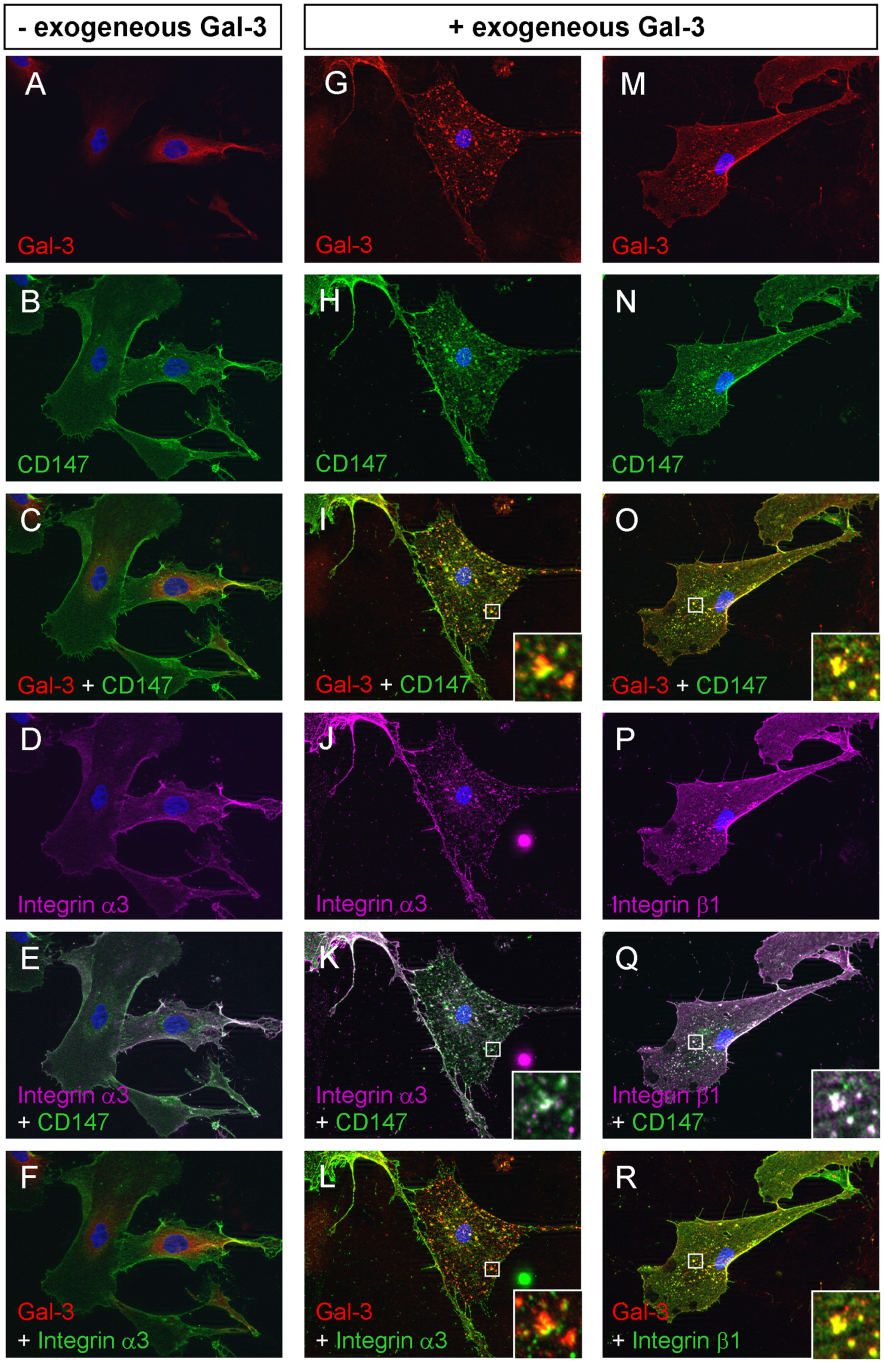
Immunohistochemical analysis of co-localization between Gal-3, CD147, integrin-α3, and integrin-β1 in dedifferentiated human RPE cells. *Left panels:* Cultured human RPE cells without prior exposure to exogenous Gal-3 show immunoreactivity for endogenous Gal-3 (**A**) throughout the cytoplasm including the perinuclear region and only weak staining of the plasma membrane, while reactivity for CD147 (**B**,**C**) and integrin-α3 (**D**,**E**,**F**) is diffusely distributed over the plasma membrane with complete co-localization (**E**, *white*) of integrin-α3 and CD147. *Middle and right panels:* Exposure to Gal-3 induces redistribution of CD147, integrin-β1 and integrin-α3. RPE cells were treated with 40 µg/mL Gal-3 for 15 minutes, fixed and stained with the same antisera. Exogenous Gal-3 binds to the RPE cell surface in a distinct punctuate pattern (**G**,**M**) and results in a marked redistribution of CD147 (**H**,**N**), integrin-α3 (**J**), und integrin-β1 (**P**) subunits into a punctuate pattern, indicative of cell membrane receptor clustering. Exogenous Gal-3 shows complete co-localization with CD147 (**I**,**O**; *yellow*) and integrin-β1 (**R**; *yellow*), while co-localization between integrin-α3 and Gal-3 (**L**; *yellow,* for better visibility purple fluorescence of integrin-α3 was converted to green) is less pronounced. The merged image of CD147 and integrin-β1 RPE cells exposed to Gal-3 also suggests a strong overlay of the two signals (**Q**, *white*). In contrast, the co-localization of CD147 and integrin-α3 (**K**, *white*) in Gal-3 treated cells was only partial. The insets are an enlarged image of the boxed regions showing discrete yellow or white spots, respectively, indicating areas of co-localization. A negative control, where the primary antibody was omitted showed no fluorescence signal (data not shown).

To corroborate the presence and distribution of CD147 on the surface of dedifferentiated RPE, cells were stained with an antibody against CD147. Fluorescence microscopy revealed prominent reactivity of the plasma membrane, consistent with even distribution all over the RPE cell surface (Fig. 3B,C). Staining of cultured RPE cells with an antibody against Gal-3 showed that the expression of the lectin was abundant intracellular and predominantly perinuclear, which is in agreement with findings in other human cell types [43], [44] (Fig. 3A). Consistent with this, the merged image of CD147 and Gal-3 reveals only very weak co-localization (yellow) of the two proteins at the RPE cell surface (Fig. 3C). Thus, in dedifferentiated RPE CD147 is evenly distributed over the RPE cell surface, which is in clear contrast to the polarized distribution found in native RPE [34].

To determine whether CD147 also co-localizes with exogenously added Gal-3 cultured RPE cells were treated with 40 µg/mL Gal-3 for 15 minutes before fixation and immunostaining. In addition to a prominent staining for CD147 at the plasma membrane cells exposed to Gal-3 exhibited a distinct punctuate staining pattern throughout the cell body (Fig. 3H,N). The distribution of Gal-3 binding strongly overlapped with CD147 (Fig. 3 I,O; *yellow*), with both entities present on the cell membrane as well as in a punctuate pattern. This was in clear contrast to the immunoreactivity of RPE cells without prior Gal-3 treatment (Fig. 3, *left panel* *s*) and could be indicative of cell membrane receptor clustering (Fig. 3, *middle and right panels*). This is supported by the finding that untreated RPE cells also show an evenly distributed immunoreactivity for integrin-α3 (Fig. 3D) on the RPE cell surface with complete overlap between CD147 and integrin-α3 (Fig. 3E, *white*). In contrast, in Gal-3 treated cells occasional clustering of integrin-α3 immunoreactivity (Fig. 3J) was apparent. However, in support of our findings from reciprocal immunoprecipitation double staining revealed only partial colocalization between Gal-3 and integrin-α3 (Fig. 3L, *yellow*) or CD147 and integrin-α3 (Fig. 3K, *white*) at the sites of receptor clustering. Integrin transmembrane receptors are always composed of α and β subunits. To corroborate, whether in case of α3β1 integrin, Gal-3 may interact with the β1 subunit, subcellular distribution was investigated. Immunocytochemistry revealed that in untreated cells, staining for integrin-β1 was similarly evenly distributed as integrin-a3 staining (data not shown), while in Gal-3 treated cells the staining pattern for integrin-β1 presented in a pronounced punctuate pattern indicating clustering (Fig. 3P). This pattern was similar to that of Gal-3 (Fig. 3G,M) and CD147 (Fig. 3H,N) with complete signal co-localization between exogenous Gal-3 and CD147 (Fig. 3 O, *yellow*), as well as between Gal-3 and integrin-β1 (Fig. 3R, *yellow*), and between integrin-β1 and CD147 (Fig. 3Q, *white*). In accordance with the results from reciprocal immunoprecipitation as well as from blocking experiments in FACS analyses, this may indicate that Gal-3 associates with α3β1 integrin via binding to integrin-β1 or CD147 and to a lesser extent via direct interaction with the α3 subunit. Controls with secondary antibody alone were negative for fluorescent signal (data not shown).

### RPE-derived CD147 and Integrin-β1 Contain a High Amount of β1,6-branched N-Glycans

**Figure 4:**
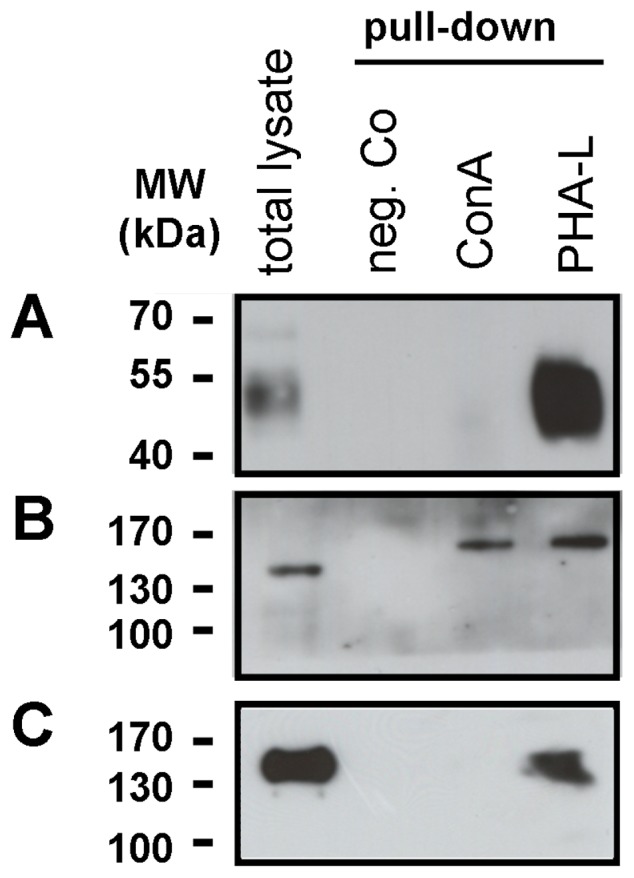
CD147, integrin-β1 and integrin-α3 in dedifferentiated human RPE cells contain β1,6-GlcNAc-branched N-glycans. Total cellular lysates were incubated with agarose-bound Concanavalin-A (ConA), a plant lectin specific for high mannose type oligosaccharides, or Phaseus vulgaris leukoagglutinin (PHA- L), which is specific for β1,6-branched N-glycans, and precipitated glycoprotein complexes were immunoblotted with either anti-CD147 (**A**), or anti-integrin-α3 (**B**), or anti-integrin-β1 (**C**) antibodies as indicated. Sepharose beads alone were used as negative control. High abundance of CD147 (**A**) and integrin-β1 (**C**) in the PHA-L-bound (specificity: β1,6-GlcNAc-N-glycans) fraction, but complete absence in eluates from ConA. (**B**) Integrin-α3 carries high-mannose type and β1,6-GlcNAc-branched N-glycans. No reactive compounds were detected in cell extracts incubated with agarose beads alone (Neg. Co; negative control). Molecular weight in kDa is indicated on the left (MW). Representative blots from four experiments are shown.

Since our experiments point to CD147 and integrin-β1 as the major Gal-3 interacting proteins at the surface of dedifferentiated RPE cells, we next sought to investigate the state of N-glycosylation state of these proteins, because binding of Gal-3 critically depends on the presence of β-1,6-N-acetyl-D-glucosamine (GlcNAc) branched glycans [13]–[15]. In order to determine whether RPE-derived CD147 and integrin-β1 contain the respective N-glycosylation patterns, pull down experiments were conducted using agarose-bound plant lectins. Previous studies have shown that leukoagglutinin of Phaseus vulgaris (PHA-L) specifically interacts with β1,6-GlcNAc-branched N-glycans [45]. Concanavalin A (ConA), which binds high mannose-type oligosaccharides, was used as a control lectin. As shown in Figure 4, reactivity for CD147 was prominent in glycoprotein complexes precipitated with PHA-L, while the binding to ConA was absent (Fig. 4A). A similar reactivity was found for integrin-β1, the other major Gal-3 binding protein. (Fig. 4C), while western blots with anti-integrin-α3 recovered both, a high-mannose type and a β1,6-GlcNAc-branched N-glycan fraction (Fig. 4B). These data suggest that CD147, integrin-α3, and integrin-β1 in RPE cells carry the preferred N-glycans for Gal-3 binding. The predominance of β1,6-branched complex N-glycans on CD147 and integrin-β1 makes them therefore suitably glycosylated for carbohydrate-dependent interaction with Gal-3.

### Localization of Exogenous Gal-3, CD147 and Integrin-β1 in Human PVR Membranes

**Figure 5:**
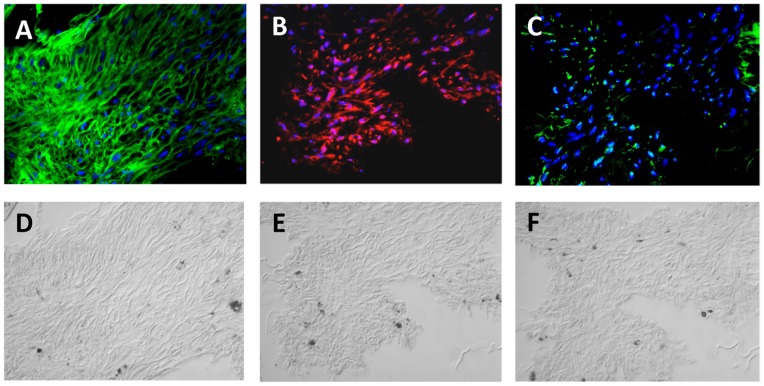
Micrographs showing immunohistochemical staining of an epiretinal PVR membrane section with biotinylated recombinant Galectin-3 (Gal-3) (A), anti-CD147 (B), and anti-integrin-β1 (C). Corresponding phase contrast images are also shown. Nuclei of cellular elements are stained with DAPI. Magnification 40x.

PVR membranes typically contain a predominance of proliferative and migratory RPE cells as well as other cell types at various stages of epithelial-to-mesenchymal transition (transdifferentiation) [20]. Having found a functional interaction of exogenous Gal-3 with CD147 and integrin-β1 in dedifferentiated RPE cells *in vitro*, we were interested whether cells in specimen of PVR may encounter exogenous Gal-3. For this purpose, paraffin sections of human PVR membranes were treated with recombinant Gal-3 and subsequently stained with an antibody specific for Gal-3. Immunohistochemistry revealed specific staining in all PVR membrane sections studied (Fig. 5A), indicative of binding of Gal-3 to the fibronectin-rich ECM found in PVR membranes. Staining for Gal-3 was present throughout the ECM of the PVR membrane, occurred in a patchy pattern, and appeared to encircle the cell bodies, so that in this setting RPE cells in PVR membranes could encounter exogenous Gal-3. In order to determine, whether the RPE-specific transmembrane counter-receptors for Gal-3, CD147 and integrin-β1, are found within PVR membranes, sections were stained for CD147 and integrin-β1. In correspondence to our in vitro findings, immunohistochemistry revealed a diffuse staining of the cell bodies for CD147 (Fig. 5B), whereas integrin-β1 appeared in a distinct and punctuate pattern (Fig. 5C). All control sections incubated without the primary antibody were unstained (data not shown). These findings suggest that an interaction between exogenously added Gal-3 and CD147 and integrin-β1 may also occur under *in vivo* conditions.

## Discussion

The overall goal of the present study was to define and validate Gal-3 interacting proteins on the surface of dedifferentiated RPE cells. This study identified CD147 and integrin-β1 as major Gal-3 interacting proteins on RPE cells and provides evidence that CD147 is a novel functional RPE transmembrane receptor for Gal-3. CD147 was isolated together with integrin-β1 by co-immunoprecipitation with Gal-3, and its identity was determined by mass spectrometry and confirmed by western blot of co-immunoprecipitation experiments. Evidence of a Gal-3/CD147 and Gal-3/integrin-β1 interaction at the RPE cell surface was further obtained from inhibition studies, where pre-incubation with anti-CD147 antibodies as well as anti-integrin-β1 antibodies abrogated binding of Gal-3 to the RPE cells. Exposure of RPE cells to exogenous Gal-3 lead to a redistribution of CD147 and integrin-β1 via interaction with Mgat5-modified N-glycans on the protein backbones, indicative of receptor clustering.

Searching for RPE-specific Gal-3 ligands by a combined approach of co-immunoprecipitation with quantitative mass spectrometry yielded a number of proteins comprising intracellular as well as extracellular located proteins. Identification of intracellular proteins most likely correlates to the abundant expression of Gal-3 in the cytoplasm and the nucleus of proliferating cells and may reflect its contribution to mRNA splicing or cell-cycle control (reviewed by Liu et al.) [46]. However, with respect to the cell surface binding of exogenous Gal-3 and its interference with ERK-MAPK activation in dedifferentiated RPE cells [29], ligands allocated to the plasma membrane were the focus of our study. Various co-immunoprecipitation experiments in different cell types have shown previously that Gal-3 associates with several cell surface glycoproteins, including MUC-1, LAMPs, the receptors for EGF, TGF-β, and VEGF, but also CD98, CD45 and CD71 [2], [12], [14], [17], [18], [47], among others. As described elsewhere in more detail, galectins can interact with integrins, namely Gal-3 was reported to interact with αvβ3, α1β1, α3β1, or α3β1 integrin in other cell types [10]–[13], [19]. The finding that in our study Gal-3 was pulled down together with integrin-α3 and integrin-β1 would fit well into the overall theme of a Gal-3/α3β1-integrin interaction [19]. However, although we used low stringency conditions in the re-immunoprecipitation experiments to avoid dissociation of the protein complexes, we could not detect significant amounts of Gal-3 in integrin-α3 eluates, and in addition we observed only partial co-localization of integrin-α3 with Gal-3 in immunocytochemistry experiments. Finally, blocking of integrin-α3 binding sites by specific antibodies failed to prevent Gal-3 binding to the RPE cell surface in flowcytometry. This was in contrast to the experimental observations for the integrin-β1 subunit: Gal-3 could be recovered from the subunit-specific integrin-β1 immunoprecipitates, blocking of integrin-β1 on RPE cell surface partially abrogated Gal-3 binding and integrin-β1 completely co-localized with exogenous Gal-3 on the RPE cell surface, suggesting that in RPE cells Gal-3 directly associates with the integrin-β1 subunit of the α3β1 integrin heterodimer, whereas the association with integrin-α3 appears to be indirect or weaker. These findings are in some contrast to a recent report by Saravanan et al. [19] showing that in a different cell type, corneal epithelial cells, Gal-3 induces lamellipodia formation via interaction with the α3 subunit of the α3β1-integrin heterodimer, exclusively. However, these discrepancies could derive from differences in the experimental approaches as well as be due to the different cell types studied. Saravanan et al. did not validate the Gal-3/integrin-α3 association by reciprocal experiments and the role of integrin-β1 in the interaction was not investigated, thus not necessarily excluding a direct interaction between Gal-3 and integrin- β1. Furthermore, it is well established, that Gal-3 influences cellular events in a cell-type specific manner, most likely dependent on varieties in the glycan profile [16].

The novel finding of the present study is that Gal-3 can associate with CD147 [48], a widely distributed, highly glycosylated 44–45 kDa cell-surface protein with two immunoglobulin domains. Gal-3/CD147 association was established by means of reciprocal co-immunoprecipitation, and confirmed by FACS analysis and cell surface co-localization experiments.

The human CD147 protein has originally been named for its extracellular matrix metalloproteinase induction (EMMPRIN) activity and is identical to the M6 leukocyte activation antigen [49]. Also, CD147 is highly homologous to a rat molecule named OX-47 [50] or CE9 [51], the mouse basigin [52] or gp42 [53] molecule, and the chicken HT7 [54], neurothelin [55] or 5A11 antigen [56]. CD147 expression is often elevated on tumor cells [57] [58], and it is implicated in various inflammatory disease states, including atherosclerosis, rheumatoid arthritis or chronic liver disease [59], [60]. CD147 on tumor cells stimulates MMP production by stromal cells, thereby leading to extracellular matrix degradation [61], elevated tumor growth and metastasis. Stimulation of CD147 promotes angiogenesis through hypoxia-inducible factor (HIF)-2α-mediated upregulation of VEGFR-2 and the soluble isoforms of VEGF in endothelial cells [62], but it can also influence angiogenesis indirectly by stimulating fibroblasts to secrete urokinase-plasminogen-activator in a paracrine fashion [63]. CD147 has also been shown to regulate lymphocyte responsiveness and it appears to be essential for cyclophilin-dependent signaling related to chemotaxis of immune cells [42] and adhesion [64]. Interestingly, a monoclonal antibody screen for α3β1 integrin-associated proteins revealed a robust association of the structurally similar α3β1 and α6β1 integrin, but not α2β1 or α5β1 integrin, with CD147. However, at present the functional relevance of this interaction is not clear yet [1]. CD147 also associates with the monocarboxylate transporters MCT-1 and -4 [34], [65] and facilitates their targeting to the plasma membrane, where they act as lactic acid transporters. Exemplary for this, CD147 is found to be associated with monocaroboxylate (MCT) transporters in the apical and basolateral membrane of healthy RPE cells. Basigin −/− null mice are blind and it has been suggested that this is due to an inappropriate targeting of the monocarboxylate transporters to the plasma membrane of the RPE and Müller glial cells, thereby leading to retinal degeneration [33]. Notably, we recovered α3-integrin and MCT-4 in Gal-3 immunoprecipitates under non-stringent conditions, but were not able to retrieve Gal-3 in reciprocal experiments using subtype specific anti-integrin-α3. Although we did not test for proteins precipitated under stringent conditions, these experiments in support with the previously shown association of CD147 with MCT-4 and integrin-α3 [1], [65] suggest, that the latter ones may have been precipitated as interactors of CD147 rather than as direct binders of Gal-3.

As a transmembrane glycoprotein, CD147 forms homo-oligomers in both heterotypic and homotypic cell-cell interactions. These homophilic associations evident as cell surface clustering of CD147 are necessary to induce MMP-production and HIF-2α-mediated regulation of soluble VEGF and VEGFR-2 expression [59], [62], [66]. The mechanisms ruling over association/dissociation of CD147 homodimers however remain largely elusive. However, there is evidence that association of caveolin-1 with CD147 leads to decreased CD147 cell surface clustering, thus inhibiting CD147-induced MMP production [3]. Caveolin-1 therefore appears to promote dissociation of CD147 homo-oligomers. The existence of such dissociation regulators implies that there might also exist receptors that trigger association of CD147 molecules. Our observation that exogenous Gal-3 induces clustering of CD147 and integrin-β1 may indicate that multivalent Gal-3 triggers cross-linking of CD147 and integrin-β1 and induces the recruitment of the two glycoproteins to receptor microdomains.

CD147 cell surface clustering and the MMP-inducing functions of CD147 are critically dependent on its level of N-glycosylation, namely on its content of complex β1,6-branched N-glycans. It has long been suggested that a lectin-like regulator of CD147 function exploiting its high level of glycosylation may exist [41]. Using PHA-L, a plant lectin with a fine specificity for β1,6-GlcNAc-branched N-glycans, we detected a high level of complex β1,6 branched N-glycans on CD147 and β1-integrin isolated from dedifferentiated RPE cells, supporting the hypothesis that Gal-3 is an inducer of CD147 clustering.

The functional relevance of Gal-3/CD147/integrin-β1 association in RPE cells is unclear. The co-localization with integrin-β1 suggests that Gal-3 may be part of the CD147/integrin-β1 complex [1]. Clustering of CD147 and integrin-β1 together with a complete overlay of the two glycoproteins upon addition of Gal-3 further underscores this assumption. So far we have found that the inhibitory effect of Gal-3 on the adhesion of dedifferentiated RPE cells to extracellular matrix was not altered by anti-CD147 antibodies, while blocking of integrin-β1 reduced both, RPE attachment and binding of Gal-3 to the RPE cell surface (unpublished data). Also anti-CD147 antibodies did not alter the attachment rate of RPE cells when compared to untreated controls, excluding a direct role of CD147 in RPE cell adhesion. Furthermore, preliminary evidence from our study suggests that the Gal-3/CD147/integrin-β1 association is not related to cell adhesion, since we also observed the glycoprotein clustering on adherent cells. This is supported by findings from Berditchevsky et al. [1], who described an interaction between α3β1 integrin and CD147, but were also not able to delineate a functional relevance of this association in cell adhesion.

However, it is perhaps more than a coincidence that Gal-3, CD147 and integrin-β1 have been linked to increased malignancy in a number of tumors [57], [58], [67]–[71], and that independently from each other all three have been found to promote angiogenesis [10], [12], [62], [72] and to influence immune responses [2], [59], [73], [74]. Clearly, further studies will be needed to determine the extent to which the physical association between CD147, integrin-β1 and Gal-3 on RPE cells may play a role in these processes with special focus on adhesion-induced metalloproteinase production and possible downstream effects triggered by this association, such as activation of Rac1 signaling, FAK, ERK, PI3K, or AKT among others [19], [59], [69], [75].

Adhesion, migration, proliferation and matrix remodeling by secretion of MMPs and synthesis of ECM components by dedifferentiated RPE cells are the key cellular events in the onset of the PVR. Since exogenous Gal-3 bound to the ECM of PVR membranes, and both CD147 and integrin-β1 could be detected in human specimen of PVR, this interaction could be of relevance in the clinical situation. Although not investigated at this point, the activity profile of the three interacting proteins identified here, together with the increased expression of Gal-3 upon RPE dedifferentiation [29], [76], would fit well into the theme of the pathogenesis of the disease. As compared with other mechanisms for affinity/avidity regulation, glycan-protein interactions may be particularly well suited for mediating the prolonged changes in cell behavior that accompany profound phenotypic alterations such as those observed during epithelial-to-mesenchymal transition of the RPE in PVR. Our findings may therefore represent a potential mechanism for glycosylation-dependent and Gal-3 mediated clustering of cell-membrane receptors in dedifferentiated RPE cells and may contribute to explain the cellular processes observed the pathogenesis of the disease. Further investigations at this pathway may lead to a better understanding of PVR and aid in development of therapeutic agents targeting the glycosylation pattern of dedifferentiated RPE cells.

## Supporting Information

Table S1 **Relative abundances of proteins identified by mass spectrometry.**
(XLS)
